# Prevalence of Low Mood, Thoughts of Self-Harm and Suicidal Ideation in Women Affected by the Perimenopause and Menopause

**DOI:** 10.1192/bjo.2024.161

**Published:** 2024-08-01

**Authors:** Olivia Hendriks, Aini Kamal, Dan Reisel, Louise Newson, Pooja Saini

**Affiliations:** 1 Liverpool John Moores University, Liverpool, United Kingdom; 2 Newson Health, Stratford-upon-Avon, United Kingdom

## Abstract

**Aims:**

Low mood is a common clinical symptom during the perimenopause and menopause. However, the extent to which low mood in menopausal women intersects with thoughts of self-harm and suicidal ideation is largely unknown. In this study we aimed to explore these questions by using two different validated symptom scores.

**Methods:**

We administered a modified version of the Greene Climacteric Symptom Questionnaire (Greene 1976) to all new patients attending the Newson Health Menopause and Wellbeing Clinic, Stratford-upon-Avon, between 1 September 2023 and 31 December 2023. Patients were also asked to complete the PHQ-9 symptom questionnaire, an instrument for diagnosing and measuring the severity of depression. Data were collected from electronic health records and analysed using descriptive statistics.

**Results:**

1,212 patients were included in the study and completed the Greene Climacteric and PHQ-9 questionnaires at baseline and after 3 months. Mood and mental health symptoms including self-reported anxiety and depression affected 98% of patients. 16% of respondents indicated that they had thoughts of self-harm or suicidal ideation on at least some days in the 2 weeks prior to their initial appointment (Question 9 of the PHQ-9).

**Conclusion:**

The findings of our study demonstrate that negative mood symptoms are common in perimenopausal and menopausal women. 1 in 6 women reported thoughts of self-harm prior to initiation of HRT. Our observational data suggest that mood symptoms are highly prevalent and some women have severe symptoms and may experience suicidal ideation. Our findings should inform better mental health support and access to treatment for women experiencing negative mood symptoms in the menopause transition.

